# Deconstructing Traumatic Mission Experiences: Identifying Critical Incidents and Their Relevance for the Mental and Physical Health Among Emergency Medical Service Personnel

**DOI:** 10.3389/fpsyg.2019.02305

**Published:** 2019-10-22

**Authors:** Alexander Behnke, Roberto Rojas, Sarah Karrasch, Melissa Hitzler, Iris-Tatjana Kolassa

**Affiliations:** ^1^Clinical and Biological Psychology, Institute of Psychology and Education, Ulm University, Ulm, Germany; ^2^University Psychotherapeutic Outpatient Clinic, Institute of Psychology and Education, Ulm University, Ulm, Germany

**Keywords:** ambulance personnel, paramedics, critical incident, secondary traumatic stress, detached concern, vicarious traumatisation, occupational stress and mental-physical health, compassion fatigue

## Abstract

Emergency medical service (EMS) personnel frequently encounter emotionally stressful or even traumatic incidents in their line of duty. In this study, a checklist of emotionally stressful events for the German EMS was introduced. A mixed-method approach was used to identify mission events that were critical for the development of mental and physical stress symptoms. Data were collected in a cross-sectional sample of 102 EMS employees. A quantitative content analysis of the participants’ worst experiences on duty indicated, traumatic missions to be a concatenation of two to five emotionally stressful events. Rescue missions were experienced as traumatic if (i) EMS personnel became victims of attacks or threats; (ii) certain circumstances caused them to give up their professional detachment from patients; (iii) EMS personnel perceived the overall mission as exceptionally tragic. In subsequent correlation analyses, the corresponding checklist items showed consistent positive associations with the post-traumatic, depressive and physical stress symptoms among the study cohort. Within the exploratory regressions, the sum score of critical on-duty exposures contributed incrementally to the prediction of mental and physical stress symptoms when non-work-related trauma exposure and perceived social support were also considered. Findings point toward the importance of considering the cumulative burden of critical incidents for the long-term health of EMS personnel. Future research is needed to investigate, how on-duty trauma affects the social support EMS personnel received from their work and personal relationships.

## Introduction

During their daily work, emergency medical service (EMS) personnel are subject to experience numerous emotionally distressing and even potentially traumatic incidents. Among them are *direct* exposures to threats of the helpers’ personal safety and *indirect* (secondary) exposures as witness of other humans’ suffering or trauma (Michael et al., 2016). Indirect exposures to critical incidents on duty are referred to as *vicarious traumatisation* and its persistent negative psychological consequences for the helpers’ health and work ability were labelled as *compassion fatigue* or *secondary traumatic stress* (STS) symptoms (Argentero and Setti, 2011; Cocker and Joss, 2016; Ludick and Figley, 2017). STS symptoms strongly correspond with post-traumatic stress symptoms; i.e., they involve symptoms of recurrent and involuntary thoughts and memories of witnessed trauma (*intrusion*), persistent emotional and situational avoidance of reminders (*avoidance*), and increased negative emotional reactivity (*arousal*) (Reinhard and Maercker, 2004). In principle, any event that evokes unusually intense aversive emotional stress can present a critical incident triggering the development of STS symptoms as well as related depressive and physical symptoms (Reinhard and Maercker, 2004; Michael et al., 2016). However, several studies attempted to identify which types of rescue missions are particularly ‘critical’ (e.g., Halpern et al., 2009). Qualitative studies postulated that critical missions are not traumatising because of the encounter with a certain type of event alone rather it is the concatenation of various emotionally stressful aspects within one mission (Alexander and Klein, 2001; Regehr et al., 2002; Van Der Ploeg and Kleber, 2003). To investigate this in detail, we conducted a quantitative content analysis of the worst work experience of our participants.

Although exposure to a single traumatic mission can put EMS personnel at risk for trauma-related symptoms, it is rather the frequent and repeated confrontation with a multitude of complex and emotionally challenging situations on duty that increases the vulnerability to develop mental and physical stress symptoms, which may ultimately consolidate as mental health disorders. Compared to the general population, all types of first responders (e.g., police officers, firefighters, emergency nurses) face an increased risk of developing trauma-associated mental disorders such as post-traumatic stress disorder (PTSD), depression, somatic disorder, or substance consumption disorder (Goldfrank et al., 2003; Benedek et al., 2007; Bryant and Guthrie, 2007; Häller et al., 2009; Berger et al., 2012; Donnelly, 2012). However, EMS personnel show even higher rates of trauma-associated mental health problems than other first responders, such as firefighters and police officers (Berger et al., 2012). Efforts were made to merge potentially stressful mission events within checklists in order to quantify the cumulative frequency of encountering stressful and critical incidents among the U.S. and Dutch EMS (Monnier et al., 2002; Declercq et al., 2011; Donnelly and Bennett, 2014). In comparison, studies in German-speaking EMS personnel used relatively incomplete event lists (Teegen and Yasui, 2000; Reinhard and Maercker, 2004; Schoch, 2008; Häller et al., 2009; Schöfmann, 2010; Döllinger, 2014). Thus, it was our aim to develop an updated checklist of emotionally stressful and critical mission incidents for the German EMS. For this, a focus group of experienced EMS personnel were formed in order to obtain key informants’ knowledge during the development process.

We expected not all stressful events that EMS personnel encounter on rescue missions to be necessarily critical for persistent negative consequences on the helpers’ health. Previous research has indicated that the emotions members of a rescue crew feel during and after missions are pivotal for the development of STS symptoms (Halpern et al., 2012). Studies analysing interview statements and free text comments from EMS personnel concluded that the most emotionally disturbing missions typically include emotional involvement with the patients (Regehr et al., 2002; Van Der Ploeg and Kleber, 2003; Jonsson and Segesten, 2004; Halpern et al., 2009, 2012; Avraham et al., 2014). Ludick and Figley (2017) characterised empathy as a ‘double-edged sword’ in any helping profession. That is, EMS personnel need to empathise with their patients at the same time they need to avoid strong emotional proximity to the patients. The balance of compassion and emotional distance is often labelled *detached concern* (e.g., Lampert and Glaser, 2018). Strong emotional proximity bears the risk of evoking aversive emotions in EMS personnel which were linked to the subsequent development of STS symptoms. Some of the exhibited emotions are intensive compassion with patients as well as anger, sadness, despair, fear, or helplessness (Jonsson and Segesten, 2004; Halpern et al., 2009, 2012; Berger et al., 2012; Avraham et al., 2014; Donnelly and Bennett, 2014; Lampert and Glaser, 2018). Research identified a number of situations in which EMS personnel are at high risk of losing their professionally detached perspective and emotionally identifying with their patients. Among these situations are rescue missions involving medical care for victims of burns, serious or fatal accidents or acts of violent crime as well as dealing with dead bodies or severed body parts, treating seriously injured or dying children, encounter patients that are family, friends or personally known to the rescue crew, and being attacked or threatened during missions (e.g., Clohessy and Ehlers, 1999; Alexander and Klein, 2001; Regehr et al., 2002; Jonsson et al., 2003; Van Der Ploeg and Kleber, 2003; Avraham et al., 2014). Therefore, we expected these events (i) to frequently appear as an aspect in EMS personnel’s worst mission experiences and (ii) to be linked to higher STS, depressive, and physical stress symptomatology.

Due to conceptual reasons authors of existing checklists avoided to include items on how EMS workers emotionally perceived rescue missions (for a discussion see Donnelly and Bennett, 2014). Interestingly, Donnelly and Bennett (2014) further asked their respondents to suggest additional aspects that were not previously included in the employed checklist. Those respondents emphasised to consider, if they have ever experienced missions which they perceived as gruesome, tragic or in which they felt helpless. As missions evoking intensive emotions in helpers were reported to be highly predictive for STS symptom incidence (Regehr et al., 2002; Van Der Ploeg and Kleber, 2003; Jonsson and Segesten, 2004; Halpern et al., 2009, 2012), our checklist included items to assess how EMS workers emotionally experienced missions. We expected these items to have a high relevance in predicting STS, depressive, and physical stress symptoms.

Aforementioned inventories assess the frequency of encountering different types of critical situations. However, it could be difficult for EMS personnel to specifically recall how often they encountered a certain event type throughout their whole career in the EMS. This might result in inaccurate responses especially from experienced EMS personnel (Donnelly and Bennett, 2014). And this could explain why the summarised frequency of encounters did not consistently correlate with EMS personnel’s mental and physical stress symptoms in previous studies (e.g., Teegen and Yasui, 2000; Reinhard and Maercker, 2004; Declercq et al., 2011; Döllinger, 2014). Methodological research among populations exposed to war-related trauma have indicated that it is not the recurrence of traumatic events rather the exposure to various types of events that predicted trauma-spectrum disorder symptoms (Wilker et al., 2015; Conrad et al., 2017). Therefore, our checklist will only assess whether EMS personnel have ever encountered certain events contained within the checklist. We aimed to test whether the cumulative number of stressful event types experienced among the EMS predicts their concurrent STS, depressive and physical stress symptomatology.

As an imminent consequence of their duty, EMS personnel are repeatedly exposed to their patients’ intense suffering. Ludick and Figley, in their holistic compassion fatigue resilience theory (2017) postulated helpers’ empathic responses to the frequent exposures to human suffering as the main cause of STS symptom development. In addition, they also suggested personnel’s exposure to critical life events unrelated to the work in the EMS as a risk factor for STS symptoms. Indeed, previous studies found more severe trauma-related stress symptoms among EMS workers who reported exposure to more potentially traumatic major life events outside of their work (Teegen and Yasui, 2000; Maunder et al., 2012; Richter, 2014; Levy-Gigi et al., 2015; Wild et al., 2016). As a resilience factor, Ludick and Figley (2017) emphasised the relevance of whether trauma-exposed professionals feel socially supported by their family, friends, colleagues, and supervisors. Indeed, a number of studies with first responders showed perceived social support to reduce the risk for detrimental mental and physical consequences of trauma exposure (e.g., Regehr et al., 2002; Aasa et al., 2005; Sterud et al., 2006; Richter, 2014; Setti et al., 2016; Wild et al., 2016; Skeffington et al., 2017). The meta-analysis by Prati and Pietrantoni (2010b) confirmed that EMS personnel who have received as well as perceived more social support showed fewer trauma-related stress symptoms and a higher quality of life. However, the perception of being supported was the stronger predictor of health than the received social support. In a longitudinal study, Wild et al. (2016) observed that over the course of 2 years, EMS personnel who perceived more social support developed fewer STS and depressive symptoms. We used the theoretical framework postulated by Ludick and Figley (2017) to investigate the utility of our checklist. Specifically, we tested whether the sum score of the checklist contributes incrementally to the prediction of our participants’ mental and physical trauma-related symptoms when exposure to non-work-related critical life events and perceived social support were also taken into account.

## Materials and Methods

### Development of the Rescue and Emergency Situation Questionnaire (RESQ)

Schoch (2008) and Schöfmann (2010) developed an initial checklist of 17 emotionally stressful mission aspects for the German EMS. However, during staff orientation events at German Red Cross ambulance stations, we repeatedly received feedback from EMS employees that Schoch’s checklist did not contain all emotionally stressful aspects of EMS missions. Given the need for revision and expansion, we invited five experienced EMS workers to submit item proposals to clarify and complete the checklist. The original and additionally proposed items were discussed with a focus group of experienced EMS personnel. Based on the results of the discussion a revised checklist was proposed. To ensure the comprehensiveness and accuracy of the revised checklist it was rereviewed by the focus group members. Eventually, the revision led to the inclusion of 14 additional aspects, which mainly included items on the care for specific patient groups (i.e., perpetrators of serious violent crimes and minor acts of violence, burn victims, patients with mental disorders, patients with mental or physical disabilities, palliative or comatose patients, refugees, homeless, or lonely patients). We further added two items related to unsuccessful resuscitations and aggressive relatives. The final checklist—called *Rescue and Emergency Situations Questionnaire* (RESQ; see Table 2)—comprised a total of 31 different mission aspects and was applied in this study. Participants were asked to indicate with *no* or *yes* on whether they have experienced any of the events listed during their work in the EMS. Additionally, participants could add events which were not included in the event list as a free text.

### The Study

#### Procedure

The study was approved by the local ethics committee and carried out in accordance with the provisions of the Declaration of Helsinki. All participants were EMS personnel from two German Red Cross ambulance stations in Baden-Württemberg, Germany. The aim and the procedures of the study were introduced during on-the-job education events to 247 of the 318 employees of the two stations. Through e-mail employees were invited to participate in an online survey (LimeSurvey GmbH, 2017). Of the 247 invited employees, 115 participated in the survey between October 2016 and April 2017, presenting a response rate of 46.6%. Prior to the survey participation all the participants declared their written informed consent. There was no remuneration for participation.

#### Sample Characteristics

For the present research question, full data was available from *N* = 102 EMS workers (65 men, 37 women). From 12 participants, responses to at least one questionnaire were missing as (i) they terminated the survey before completion or (ii) the survey was automatically terminated due to inactivity. Participants with complete and incomplete responses did not differ with regard to age, sex, work experience, work location, qualification, form of employment, quantitative work load, and exposure to stressful or critical events (see Supplementary Table 1). Regarding the level of qualification, the sample included 61 (59.8%) *Notfallsanitäter* (cf. Emergency Medical Technician–Paramedics), 11 (10.8%) *Notfallsanitäter* trainees, and 30 (29.4%) *Rettungssanitäter* (cf. Emergency Medical Technician–Basics). The sample corresponded well to the employee population in terms of work location, form of employment, sex, age, and work experience in the EMS (see Table 1). Participants were slightly younger than the entire employee population because the volunteering medical students among EMS personnel were underrepresented.

**TABLE 1:** Comparison of sample and work population of regional emergency medical service (EMS) employees.

	**Entire EMS employees**		**Study sample**		**Test statistic**
	**Absolute**	**Relative**	**Absolute**	**Relative**	
Total	318	100.0%	102	32.1%^#^	
**Work location**					χ^2^(1) = 0.00, *p* = 1.000, *V* = 0.00
Ulm station	223	70.1%	72	70.6%	
Heidenheim station	95	29.9%	30	29.4%	
**Employment**					χ^2^(2) = 11.84, *p* = 0.003, *V* = 0.17
Salaried	198	62.3%	77	75.5%	
Voluntary	101	31.8%	15	14.7%	
In apprentice	19	6.0%	10	9.8%	
**Sex**					χ^2^(1) = 0.71, *p* = 0.399, *V* = 0.05
Men	222	69.8%	66	64.7%	
Women	96	30.2%	36	35.3%	
	***M* (*SD*)**	***Mdn* (IQR)**	***M* (*SD*)**	***Mdn* (IQR)**	
Age (years)	32.1 (11.2)	27.3 (15.6)	30.1 (11.0)	26.0 (17.0)	*U* = 13514.5, *z* = −2.53, *p* = 0.011, *r* = −0.12
Years working in EMS (years)	5.7 (5.5)	3.7 (7.2)	7.7 (8.8)	3.3 (10.9)	*U* = 15362.0, *z* = −0.67, *p* = 0.506, *r* = −0.03

**^#^*Proportion of total staff. Frequency distributions of staff and sample were compared using Pearson χ^2^-tests (applying a Yates correction for 2×2-tables) with Cramer’s *V* as effect-size measure. Age and the work experience in the EMS were compared using Mann–Whitney *U*-tests with Cohen’s *r* as effect-size measure.*

From the online survey, responses from 40 participants indicated a possible mental disorder. With these 40 participants, for in-depth clinical diagnostics, clinically trained staff conducted the Structured Clinical Interview for DSM-IV (adapted for DSM-5; Wittchen et al., 1997) as well as the Clinician-Administered PTSD Scale for DSM-5 (Schnyder et al., 2013). Fifteen of them were diagnosed with one or two current mental disorder(s), while *n* = 9 met the criteria for remitted mental disorders. At the time of the study, *n* = 8 (7.0%) had a depressive disorder, *n* = 5 (4.3%) had alcohol and/or cannabis consumption disorder, and *n* = 4 (3.5%) had PTSD, i.e., one case of full PTSD, two cases of partial PTSD and a patient who refused final diagnosis.

#### Measures

Depressive symptoms were assessed using the German Patient Health Questionnaire scale for depression (PHQ-9; Löwe et al., 2002). On a four-point Likert scale ranging from 0 (*not at all*) to 3 (*almost every day*), participants indicated the extent to which they felt affected by nine depressive symptoms (e.g., “tiredness or feeling of no energy”) during the past 2 weeks. All items were aggregated to a sum score (Cronbach’s α = 0.84).

Symptoms of PTSD were assessed using the German PTSD Checklist for DSM-5 (PCL-5; Krüger-Gottschalk et al., 2017). Participants described the worst incident they have ever experienced on a free-text item. With regard to this index event, participants then reported how much they felt impaired by symptoms of intrusions, hyperarousal, avoidance, and negative alterations in mood or cognition during the last month. Participants responded to 20 items on a five-point Likert scale ranging from 1 (*not at all*) to 5 (*very strong*). Responses were aggregated to a sum score (Cronbach’s α = 0.91).

Physical symptoms associated with stress were assessed using the German Patient Health Questionnaire scale for physical symptoms (PHQ-15; Löwe et al., 2002). On 13 items, participants indicated how much they felt impaired by physical symptoms during the last 4 weeks (e.g., stomach aches). The item for menstrual pain was excluded to avoid gender bias. Responses were recorded on a three-point Likert scale from 0 (*not at all*) to 2 (*very strong*) and aggregated to a sum score (Cronbach’s α = 0.84) including two additional items of the PHQ-9 covering sleep disturbances within the last 2 weeks.

The German Life Events Checklist for DSM-5 (LEC-5; Ehring et al., 2014) was used to assess whether participants experienced or witnessed 16 potentially traumatic event types which included exposure to natural disasters, accidents, interpersonal violence, war, life-threatening illness, or injury. An additional free text item asked for other events not included in the event list. As the RESQ was used, the response option of being confronted with the events in the line of duty was excluded.

To measure perceived social support, the F-SozU K14 was used (Fydrich et al., 2009). On a five-point Likert scale ranging from 1 (*does not apply*) to 5 (*applies exactly*), participants responded to 14 statements regarding their emotional support, practical support, and social integration (e.g., “I get a lot of understanding and security from others”). Answers were aggregated to a sum score (Cronbach’s α = 0.95).

### Statistical Analyses

#### Quantitative Content Analysis

We aimed to investigate which aspects a traumatic mission were consisted of, and for this, participants were requested to describe their worst life experience as index event in the PCL-5 (Krüger-Gottschalk et al., 2017). These descriptions were analysed in a quantitative content analysis. As a first step, eight independent coders with clinical experience excluded all index-event descriptions referring to the private life of participants (*n* = 39, 38.2%) or that were ambiguous or incomprehensible (*n* = 10, 9.8%), leaving a total of 53 (52.0%) descriptions of duty-related worst life events for further analysis. All duty-related worst life events were indirect trauma exposures, according to DSM-5, including three direct trauma exposures (i.e., EMS workers themselves were threatened or injured). As a next step, the coders examined which emotionally stressful mission aspects (listed in the RESQ) were part of the respective description of the participants’ worst duty-related life event and they were coded as “1” whereas any uncontained mission aspects were coded “0.” Multiple mission aspects could be coded within one worst duty-related life event. The authors A.B. and R.R. independently corrected non-instructional coding and objective coding errors. Disagreements were solved by consensus. Coding objectivity was determined for each mission aspect using Krippendorff’s α (Krippendorff, 2011). Alpha values ≥ 0.80 indicated a sufficiently high intercoder agreement. Classification and data aggregation were performed in Microsoft Excel 2010 applying Jenderek’s (2006) macro for Krippendorff’s α.

#### Correlation and Regression Analyses

Statistical analyses were done in R 3.5.1 (R Core Team, 2018). Correlations were computed to analyse the statistical association between single checklist items (i.e., encountered mission aspects) and the outcome variables (i.e., depressive, post-traumatic, and physical symptoms). Point biserial correlations (*r*_pb_) were computed because of the RESQ items’ dichotomy. Statistical associations between single checklist items and symptom severity were expected to be small in size and not necessarily pass the threshold of statistical significance. Therefore, we considered checklist items showing a consistent positive relationship to all symptom types with at best *r*_pb_ ≥ 0.10 the most relevant and summarised them to the RESQ *critical exposure* (RESQ-CE) score.

Associations among the study variables were examined using Spearman rank correlations (*r*_S_) to account for non-normal variable distributions. Using linear regression analyses, we explored the incremental value of the RESQ and RESQ-CE scores as a statistical predictor of the study cohort’s STS, depressive, and physical symptoms while also considering non-work-related critical life events and perceived social support as predictors in the same regression model. As recommended by Field and Wilcox (2017), robust MM-estimator based linear regressions using the *MASS* package by Ripley et al. (2019) were computed in case of non-normally distributed model residuals.

## Results

### Aspects Shaping Traumatic Medical Emergency Missions

In order to investigate the aspects which constituted a traumatic mission, a quantitative content analysis was conducted on 53 descriptions of the EMS personnel’s worst duty-related life events. The content analysis was highly objective as indicated by the intercoder agreement (Krippendorff’s α = 0.80–0.99, see Table 2). The analysis revealed that traumatic medical rescue missions were highly diverse in regard to the types of events EMS personnel encountered. However, traumatic missions did not consist of a single stressful mission aspect but rather a combination of *M* ± *SD* = 2.6 ± 1.5 mission aspects. The most common aspects were: the care for victims of road accidents (40%), unsuccessful resuscitations (27%), caring for seriously or fatally injured children (25%), victims of severe violent crimes (17%), and victims of suicide or attempted suicides (13%) as well as being confronted with severed body parts (13%). Notably, EMS personnel typically described their worst life events on duty as particularly tragic. Tragedy was experienced mostly due to the accident’s circumstances (21%), background information about the twist of fate affecting the patients (12%) or the unexpected intensity of impressions at the mission site (18%). Furthermore, EMS personnel described that they could not successfully help due to a lack of technical and/or staff resources (11%).

**TABLE 2:** Checklist items with results of quantitative content and correlation analyses.

**No.**	**Item**	**Proportion of participants who reported event**	**Coding frequency in content analysis (*N* = 53)**	**Intercoder agreement (Krippendorff’s α)**	**Point biserial correlations (*N* = 102)**		
					**Post-traumatic symptoms (PCL-5)**	**Physical symptoms (PHQ-15)**	**Depressive symptoms (PHQ-9)**
1	A road accident in which people were trapped or severely injured	83.3%	21.13	0.99	0.04	0.02	0.07
2	An operation where child(ren) suffered life-threatening or fatal injuries	55.9%	13.13	0.99	0.07	0.05	0.06
3	Taking care of victims of severe violent crimes (e.g., murder, rape)	52.0%	9.00	1.00	−0.04	0.10	0.07
4	Taking care of victims of minor crimes (e.g., affray, stabbing)	87.3%	2.00	1.00	0.08	0.02	0.04
5	Taking care of offenders of severe violent crimes (e.g., rapist, murderer)	28.4%	3.00	1.00	−0.12	0.14	0.04
6	Taking care of offenders of minor crimes (e.g., people who beat someone up or who injured someone with a knife)	71.6%	1.00	1.00	−0.10	0.12	0.11
7	An operation where the patient suffered severe burns	44.1%	1.00	1.00	0.16	0.16	0.23
8	An operation where cardiopulmonary resuscitation efforts remained unsuccessful	93.1%	14.13	0.93	−0.01	−0.07	−0.03
9	An operation that included the treatment of mentally ill patients	99.0%	5.00	1.00	0.10	0.08	−0.10
10	An operation that included an interaction with mentally or physically disabled patients	99.0%	0.00	1.00	0.10	0.08	−0.10
11	An operation that included an interaction with people at the end of life (e.g., palliative patients)	96.1%	4.00	1.00	0.14	0.17	0.03
12	An operation that included an interaction with chronically critically ill patients (e.g., a persistent vegetative patients)	97.1%	2.00	1.00	0.01	0.12	0.05
13	An operation that included an interaction with refugees	97.1%	1.00	1.00	0.05	0.04	−0.04
14	An operation that included an interaction with people facing existential emergencies (e.g., homelessness or unemployment)	92.2%	0.00	1.00	0.09	0.06	0.02
15	An operation that included an interaction with people who were not supported adequately by their relatives	96.1%	1.25	0.83	−0.01	0.13	0.07
16	An operation that included the bearing of bad news to a patient’s relatives	74.5%	2.50	0.88	−0.16	0.05	0.02
17	An operation that included the interaction with relatives of a patient who were very demanding or aggressive	86.3%	2.25	0.89	−0.11	0.12	−0.01
18	An operation that included confrontation with severed body parts or corpse (e.g., train accidents, amputation)	60.8%	7.00	1.00	0.02	0.03	−0.03
19	An operation that included an interaction with a person who attempted or committed suicide	83.3%	7.00	1.00	0.13	0.03	0.07
20	An operation that included an interaction with a patient who was a friend of yours	51.0%	0.00	1.00	0.09	0.21	0.18
21	An operation that included the interaction with a patient whose family you were personally acquainted with	45.1%	2.00	1.00	0.04	0.16	0.16
22	An operation where you identified yourself with the victim or his/her family (e.g., dead child the same age as your own child)	42.2%	3.13	0.96	0.27	0.21	0.24
23	A case that was extremely tragic due to the accident’s sequence of events (e.g., a father chopped trees in the forest and one of the branches fell on his own daughter)	47.1%	11.25	0.84	0.29	0.17	0.23
24	A case that was extraordinarily tragic because of the twists of fate which affected a family (e.g., attending to a dying child whose family have already lost another child)	55.9%	6.13	0.98	0.10	0.16	0.07
25	An operation in which you or a colleague did not act in an optimal way (subjective opinion)	85.3%	3.88	0.97	−0.03	0.11	0.11
26	An operation in which the suboptimal actions of yours or of a colleague lead to negative consequences (objective fact)	40.2%	2.38	0.88	−0.03	0.11	0.11
27	An operation in which you were not able to help the way you wanted to (because of reasons like self-protection or human/technical resources)	48.0%	5.75	0.91	0.11	0.14	0.11
28	A case with multiple (>5) injured or dead people (Mass Casualty Incident)	65.7%	3.00	1.00	−0.13	−0.13	−0.04
29	A case that was far more tragic than it was assumed on the basis of briefing received from the rescue coordination centre	83.3%	9.75	0.80	0.07	0.17	0.09
30	An operation where you were attacked or yourself got injured	41.2%	3.00	1.00	0.19	0.29	0.22
31	An operation where a colleague was severely injured or lost his/her life	3.9%	0.00	1.00	0.03	0.07	0.00

### Prevalence of Stressful Mission Aspects Assessed With the RESQ

All stressful mission aspects were experienced by EMS personnel as part of their work (see Table 2). The majority of EMS workers (92–99%) experienced missions which involved (i) caring for patients, who were mentally ill or disabled; (ii) chronically critically ill or palliative patients; (iii) patients who could not be successfully resuscitated, and (iv) patients in social emergencies such as refugees or homeless as well as patients without adequate support from relatives. In the context of crime, a majority provided medical help to victims of criminal offences or violent crimes (72–87%). Regarding accidents, 83% of the participants were confronted with injured, trapped or killed victims of road accidents (83%) or mass casualty incidents (66%) and severed body parts (61%). Further, 83% encountered completed or attempted suicides. 83% indicated the experience of missions were much more tragic than expected as compared to the information received from the mission control centre. Most participants had to deliver bad news (75%) to the relatives or were confronted with demanding or even aggressive behaviour (87%) from relatives of the patients.

### Statistical Relationship Between Checklist Items and Stress Symptoms

**FIGURE 1:**
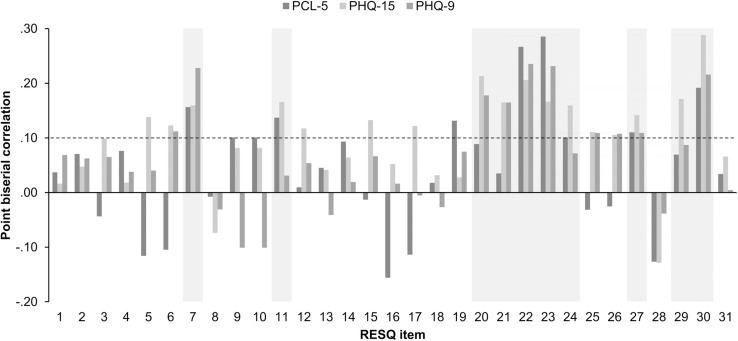
Point biserial correlations between the RESQ items and the EMS personnel’s post-traumatic (PCL-5), physical (PHQ-15), and depressive (PHQ-9) stress symptoms. The dashed line indicates a correlation of 0.10 which was defined as minimal threshold of item relevance. Items displayed on grey background were considered as the most relevant and were summarised to the RESQ-*critical exposure* score.

Figure 1 and Table 2 display point biserial correlations (*r*_pb_) between each checklist item and the participants’ STS, depressive, and physical symptoms. The items numbered 7, 11, 20–24, 27, 29, 30 showed the relatively highest and most consistent positive correlations with mental and physical symptoms. These items correspond to the following mission aspects: (i) encountering patients with severe burns (item 7); (ii) dying or palliative patients (item 11); (iii) patients that are friends or personally known to EMS personnel (item 20); (iv) having a personal relationship with the patient’s family (item 21); (v) having emotionally identified with patients and their family due to similarities with their own family (item 22); (vi) considering the occurrence of accident as particularly tragic (item 23); (vii) having background information rendering the incident extraordinary tragic (item 24); (viii) not being able to help due to lack of staff or technical resources (item 27); (ix) being unprepared for the tragedy at the mission site (item 29); and (x) being threatened or even injured during a mission (item 30). In contrary to our expectations, the items corresponding to encountering seriously injured or dying children (item 2), victims of (attempted) suicides (item 19), and bearing bad news to relatives of patients (items 16) showed no consistent and relevant sized associations with the participants’ stress symptomatology.

### Results for the RESQ and RESQ-CE Sum Score

Responses were summarised to create the RESQ score presenting the number of all emotionally stressful mission aspects encountered while working in the EMS. Across their career, EMS workers experienced on average *Mdn (IQR)* = 22.0 (6.2) emotionally stressful mission aspects (range: 0–31). The longer participants worked in the EMS the higher the number of emotionally stressful mission aspects encountered. Based on the correlation pattern examined above, the checklist items 7, 11, 20–24, 27, 29, and 30 were summarised as RESQ-*critical exposure* (RESQ-CE) score presenting the number of encountered critical (i.e., potentially traumatic) mission aspects while working in the EMS. Participants encountered on average *Mdn (IQR)* = 5.5 (5.0) critical mission aspects (range: 0–10). Higher work experience in EMS correlated with encountering more critical mission aspects.

Table 3 displays descriptive statistics and correlations of the study variables. Small to medium sized correlations between the RESQ or RESQ-CE scores with the LEC-5 sum score indicated a partial overlap of the two checklists. That is, certain life events might be recorded on both checklists, e.g., medical help to injured/dying family members or friends. Neither the RESQ nor the RESQ-CE score correlated with perceived social support. The RESQ score was positively associated with physical stress symptoms, but was only descriptively associated with post-traumatic and depressive symptoms. Conversely, the RESQ-CE score showed significant medium-sized associations with all symptom types. As expected, EMS personnel’s STS symptomatology also largely depended on lower perceived social support and the number of critical life events encountered in private life.

**TABLE 3:** Descriptive statistics and Spearman rank correlations.

	***Mdn* (*IQR*) [range]**	**2**	**3**	**4**	**5**	**6**	**7**	**8**	**9**
RESQ	22.0 (6.2) [0.0, 31.0]	0.888^∗∗∗^	0.219^∗^	0.683^∗∗∗^	–0.026	0.124	0.228^∗^	0.166	0.178
RESQ-CE	5.5 (5.0) [0.0, 10.0]	–	0.293^∗∗^	0.562^∗∗∗^	–0.094	0.314^∗∗^	0.359^∗∗∗^	0.294^∗∗^	0.359^∗∗∗^
LEC-5	4.0 (4.0) [0.0, 16.0]		–	0.106	–0.161	0.243^∗^	0.221^∗^	0.271^∗∗^	0.268^∗∗^
Years working in EMS	3.1 (10.7) [0.1, 35.0]			–	–0.094	0.131	0.118	0.123	0.132
Perceived social support	8.6 (1.8) [0.0, 10.0]				–	–0.292^∗∗^	−0.236^∗^	–0.409^∗∗∗^	–0.371^∗∗∗^
PCL-5	6.5 (12.0) [0.0, 39.0]					–	0.576^∗∗∗^	0.561^∗∗∗^	0.794^∗∗∗^
PHQ-15	5.0 (6.3) [0.0, 21.0]						–	0.773^∗∗∗^	0.899^∗∗∗^
PHQ-9	3.0 (6.3) [0.0, 18.0]							–	0.895^∗∗∗^
Symptom composite^†^	−0.3 (1.3) [−1.1, 2.6]								–

*^†^Average of *z*-standardised symptom sum scores. ^∗^*p* < 0.050, ^∗∗^*p* < 0.010, ^∗∗∗^*p* < 0.001, two-tailed, *N* = 102.*

Table 4 summarizes the results of the linear regression analyses exploring the incremental value of the RESQ and RESQ-CE scores as a statistical predictor of mental and physical stress symptoms, i.e., the exposure sum scores offer an additional improvement of the statistical prediction of the stress symptoms over non-work-related life event exposure and perceived social support. The cumulative experience of more emotionally stressful mission aspects during work in the EMS (RESQ score) was associated with higher overall symptom severity as well as higher severity of depressive and physical symptoms. However, the severity of the participants’ post-traumatic stress symptoms did not depend on the RESQ score. Conversely, higher cumulative exposure to critical incidences (RESQ-CE score) was a significant predictor for more severe mental and physical symptoms. Analyses further confirmed that the perceived social support was linked to fewer stress symptoms whereas the number of experienced non-work-related potentially traumatic life events was no significant predictor.

**TABLE 4:** Results of linear regression analyses.

	**Post-traumatic stress symptoms (PCL-5)^♢^**		**Physical symptoms (PHQ-15)**		**Depressive symptoms (PHQ-9)**		**Symptom composite^†^**	
RESQ	0.099	–	0.215^∗^	–	0.192^∗^	–	0.197^∗^	–
RESQ-CE	–	0.189^∗^	–	0.304^∗∗^	–	0.255^∗∗^	–	0.308^∗∗∗^
LEC-5	0.106	0.046	0.114	0.071	0.090	0.055	0.121	0.075
Perceived social support	−0.261^∗^	–0.290^∗∗∗^	−0.213^∗^	−0.192^∗^	–0.476^∗∗∗^	–0.458^∗∗∗^	–0.370^∗∗∗^	–0.350^∗∗∗^
*F*(3,98)	3.67^∗^	7.14^∗∗∗^	4.23^∗∗^	6.08^∗∗∗^	12.97^∗∗∗^	14.68^∗∗∗^	8.49^∗∗∗^	11.39^∗∗∗^
*R*^2^	0.101	0.111	0.114	0.157	0.284	0.310	0.206	0.258

*Separate regression analyses were computed for each outcome variable (columns), with using either the RESQ or the RESQ-CE score as predictor in addition to the influences of non-work-related potentially traumatic life events (LEC-5) and perceived social support. ^♢^MM-estimator based robust regressions were performed to account for non-normal model residuals. ^†^Average of *z*-standardised symptom sum scores. Standardised regression coefficients are displayed, ^∗^*p* < 0.050, ^∗∗^*p* < 0.010, ^∗∗∗^*p* < 0.001, two-tailed, *N* = 102.*

## Discussion

Our main objective was to develop a checklist that allows quantifying the cumulative exposure to stressful and critical events experienced by German EMS personnel in rescue missions. Unlike previous checklists, we not only sampled objective stressful event types but also included items on the emotional experience of EMS personnel. Each of the RESQ’s 31 items was endorsed by participants indicating the items’ general appropriateness of inclusion in the inventory. The RESQ sum score—i.e., the cumulated number of encountered stressful event types—did not consistently correlate with the post-traumatic, depressive, or physical stress symptoms among our participants. This finding was not unexpected as there are a number of previous studies reporting the fragile associations between the frequency of event exposure and trauma-related stress symptoms in EMS personnel (Teegen and Yasui, 2000; Reinhard and Maercker, 2004; Häller et al., 2009; Declercq et al., 2011; Döllinger, 2014). The RESQ sum score was significantly associated with the severity of the participants’ depressive and physical stress symptoms while the influences of non-work-related potentially traumatic events and perceived social support were statistically controlled.

A possible reason for the inconsistent relationship between cumulative event exposure (either assessed with previous or our current checklists) and participants’ trauma-related stress symptoms could be that not all mission events perceived as stressful were necessarily traumatic or critical for the further development of stress symptoms. Therefore, our second objective was to investigate which event types were composed of our participants’ traumatic experiences on duty and also whether these event types were associated with more severe STS, depressive, and physical symptoms among our sample of EMS personnel. Based on the literature, we expected traumatic missions to be characterised by exposures to direct threats or attacks during missions (e.g., Michael et al., 2016) and by encounters with patients or situations that are likely to trigger the empathic involvement of EMS workers with patients (e.g., Ursano et al., 1999). We also expected the EMS personnel’s overall emotional experience of the mission to be of critical importance (e.g., Halpern et al., 2012). As a second step, we tested whether particularly those checklist items that assess the exposure to the aforementioned events showed the strongest correlations with participants’ stress symptomatology.

### What Makes a Rescue Mission Traumatic?

In general, our content analysis indicated that traumatic experiences in the EMS were characterised by a concatenation of several emotionally challenging events within one rescue mission. This findings suits results of previous studies (Alexander and Klein, 2001; Regehr et al., 2002; Van Der Ploeg and Kleber, 2003). The precise occasions of traumatic rescue missions were heterogeneous. In line with previous studies, these were mainly rescue operations for victims of severe traffic accidents, for victims of violent crimes or for seriously injured and/or abused children. Traumatic missions often covered unsuccessful resuscitation attempts or confrontations with dead bodies and severed body parts. We matched these results with the statistical association of the respective mission event types with our participants’ mental and physical stress symptomatology.

Attending to victims of severe traffic accidents, to victims of violent crimes, to resuscitated patients with medical emergencies or encountered dead bodies were not statistically predictive for EMS personnel’s mental and physical stress symptoms. This could be due to the fact that EMS workers often face such events (e.g., Teegen and Yasui, 2000; Declercq et al., 2011). The low variability of responses could have limited the items’ statistical predictive value. Moreover, frequently, EMS personnel successfully rescue, stabilise and transfer their patients to further medical treatment. Success strengthens the EMS personnel’s conviction to fulfil a meaningful and highly significant duty which also promotes their self-worth and self-efficacy expectation. Related concepts such as the sense of coherence were found to promote resilience and positive psychological adaptation processes such as post-traumatic growth in EMS personnel after critical experiences (Streb et al., 2014; Behnke et al., 2019; Ragger et al., 2019). It was therefore suggested that the general situational ‘occasion’ of a rescue mission (e.g., a traffic accident) is much less important for the mission’s traumatic nature than the emotional experiences of the helpers during and after the mission (see Halpern et al., 2012 for further details).

Based on our quantitative content analysis, we found that EMS personnel elicit intense aversive emotions when personnel knew their patients personally or knew relatives of their patients, or when the patients reminded personnel of their own family members or themselves. Such circumstances are known to create a high risk for EMS personnel to lose their professionally detached perspective on the events and become empathetically involved with their patients (Ursano et al., 1999; Alexander and Klein, 2001; Regehr et al., 2002; Reinhard and Maercker, 2004; Avraham et al., 2014). This involvement is known to trigger intense aversive feelings in EMS personnel, which form the traumatic character of the rescue mission (Regehr et al., 2002; Halpern et al., 2009, 2012; Avraham et al., 2014; Ludick and Figley, 2017). In accordance with the results of our quantitative content analysis, the corresponding checklist items (20, 21, 22) showed consistent statistical associations with the mental and physical stress symptoms among the study cohort.

The content analysis further showed that EMS personnel felt incorrectly or incompletely briefed by the mission control centre and could not mentally prepare for the extent of human suffering they were confronted with at the scene of traumatic missions. Our participants described that certain traumatic missions were not successful because of avoidable circumstances such as a lack of technical or human resources which hindered EMS personnel from providing optimal help. Such circumstances are known to cause the personnel to lose their professionally detached perspectives during missions (Halpern et al., 2009; Declercq et al., 2011; Avraham et al., 2014). Due to this, aversive emotions spread among rescue crew members such as fear, hope- and helplessness but also anger and despair (Alexander and Klein, 2001; Jonsson and Segesten, 2004; Halpern et al., 2009, 2012; Avraham et al., 2014). Furthermore, it is speculated whether such events stimulate self-blaming thoughts and counterfactual ruminations in the aftermath of missions which were found to facilitate the development of mental and physical stress symptoms in EMS personnel (Richter, 2014; Wild et al., 2016; Gärtner et al., 2019). Correspondingly, the respective checklist items (27, 29) consistently correlated with more severe STS, depressive and physical stress symptoms.

In traumatic missions, EMS personnel experienced the circumstances of the accident, the general life situation, and the fate of their patients or their families as particularly tragic. To overcome the limitations of previously available event checklists (e.g., Donnelly and Bennett, 2014), we deliberately included items to record the EMS workers’ emotional evaluation of missions. In line with our expectations, these items (23, 24) showed highly consistent correlations with increased STS, depressive and physical stress symptoms.

In line with previous studies, the worst experiences of our participants further comprised encounters with injured or dead children, among them were children who became victims of deliberate maltreatment or neglect (e.g., Declercq et al., 2011). In contrast to our expectations, there were no consistent associations between encountering heavily or fatally injured children and increased stress symptomatology in this cohort of EMS personnel. Previous studies using exposure checklists for U.S. EMS personnel yielded similar results (Monnier et al., 2002; Halpern et al., 2012; Donnelly and Bennett, 2014). This and abovementioned studies have not elaborately assessed encounters with injured or dying children. Based on the qualitative feedback of their participants, Donnelly and Bennett concluded a more detailed assessment of incidents with children as necessary (see Donnelly and Bennett, 2014 for suggestions). In par with this, our participants also suggested inclusion of additional items on children as patients, e.g., encounters of sudden infant death. Future efforts to quantify stressful event exposures in EMS might thus profit from including more elaborate items.

Based on the findings of previous studies (e.g., Clohessy and Ehlers, 1999), encountering patients with severe burns was expected as a frequent aspect of participants’ traumatic missions; however, our content analysis did not confirm this. In our checklist, 44% of the study cohort, who reported to have encountered patients with severe burns, also showed increased mental and physical stress symptoms. In subsequent diagnostic interviews, EMS personnel further explained they found severe burn cases emotionally disturbing as they have to witness patients suffering in full consciousness, patients’ uncertain chances of survival as well as the high risk of infection the personnel could pose to the patient. They also found the burn injuries and burn stench highly disturbing. In addition, providing medical care for palliative and dying patients showed statistical correlations with the symptom burden of EMS personnel. Qualitative studies explored emotional difficulties associated with missions in which saving patients were impossible or very unlikely and these difficulties include guilt, anger or concerns about professional competences (Halpern et al., 2009; Avraham et al., 2014). Our content analysis also indicated that in the context of resuscitating terminally ill patients, EMS personnel are often confronted with ethical and legal ambiguities which further cause them severe emotional stress.

Our results support previous findings which have postulated helpers’ excessive compassion with patients as the core mechanism for the incidence of STS as well as depressive and physical stress symptoms (Ursano et al., 1999; Alexander and Klein, 2001; Regehr et al., 2002; Van Der Ploeg and Kleber, 2003; Jonsson and Segesten, 2004; Halpern et al., 2009, 2012; Avraham et al., 2014; Ludick and Figley, 2017; Lampert and Glaser, 2018). However, beside indirect traumatic encounters some of the EMS personnel’s worst experiences also included direct threats to their own health or lives. This was especially the case when rescue crews arrived at the scene before the area was secured by the police. Moreover, EMS workers’ lives were endangered whenever they have tried to prevent people from committing suicide in dangerous locations such as bridges or train tracks. The corresponding checklist item (30) showed by far the highest association with mental and physical stress symptoms. Indeed, direct traumatisation is regarded as a stronger risk factor for the development of post-traumatic, depressive and anxiety symptoms than the indirect traumatisation induced by compassion with patients (Reinhard and Maercker, 2004; Michael et al., 2016). In correspondence with previously reported prevalence rates, a total of 40% of this sample of EMS personnel have encountered threats or were even seriously injured during rescue missions (Teegen and Yasui, 2000; Suserud et al., 2002; Häller et al., 2009; cf. Declercq et al., 2011). Among all first responders, EMS personnel are the most likely victims of violent attacks by patients, relatives or uninvolved bystanders. The increasing propensity for violent attacks on rescue workers, as observed in Germany in recent years, highlights the particular relevance of direct traumatisation for EMS personnel’s health (Feltes and Weigert, 2018).

### The RESQ-CE Score as a Measure of Cumulative Critical Exposure in the Line of Duty

As discussed above, the number of all emotionally stressful events encountered on duty (RESQ sum score) showed a rather fragile association with the STS symptoms of EMS personnel. This was probably the case because not all events the EMS personnel perceive as stressful are necessarily traumatic. Based on the combined results of content and statistical analyses, we thus summarised the checklist’s most influential items as RESQ-CE score presenting the cumulated number of *critical* exposures in the EMS. Bivariate correlation and multiple regression analyses confirmed that the RESQ-CE score was consistently linked to higher mental and physical STS symptomatology in this sample of EMS personnel. It provided an incremental contribution to explaining the concurrent post-traumatic, depressive and physical stress symptoms when non-work-related trauma exposure and perceived social support were considered. Our results suggest the RESQ-CE score as a potential measure to retrospectively quantify cumulative critical exposures in the EMS. However, future studies in independent samples are needed to confirm the predictive value of the proposed score.

As predicted, the mental and physical stress symptoms of EMS personnel were also correlated with the number of negative major life events encountered outside of their work. This finding complements previous evidence of elevated levels of stress symptoms in EMS personnel who experienced more non-work-related critical life events. These events include serious illnesses or sudden deaths of caregivers, experiences of violence (such as physical assaults or robberies), serious accidents, childhood maltreatment experiences as well as divorce and job loss (Teegen and Yasui, 2000; Maunder et al., 2012; Richter, 2014; Levy-Gigi et al., 2015).

This study additionally confirmed the hypothesis that EMS personnel showed fewer STS symptoms when they perceived more social support from significant others. Prati and Pietrantoni (2010b) provided meta-analytical evidence for the stable association of received and perceived social support with a better overall health and life quality among EMS personnel. In this sample, perceived social support did not correlate with the number of stressful or critical encounters in the line of duty. However, frequent stressful encounters might affect the supportive social network of EMS personnel in other ways. There is evidence that the more critical incidents they encountered in the line of duty, the lesser social support they receive (Prati and Pietrantoni, 2010a). Moreover, first responders who experienced life-threatening situations on duty reported a lower need for social support (Richter, 2014). Correspondingly, Häller et al. (2009) reported that a majority of EMS personnel avoid speaking with their colleagues about emotionally burdensome experiences. On the one hand, not to show personal weakness and to avoid possible consequences of supposed mistakes; on the other hand, not to emotionally burden their colleagues and not to cause any further burden at their work environment. These observations admonish the danger that critical encounters in the EMS could lead to increasing social isolation of the personnel. Considering the high relevance of social support for EMS personnel’s health, priority should be given to improve or at least stabilise their supportive social networks. As EMS personnel particularly benefit from support by their colleagues and supervisors (e.g., Setti et al., 2016), establishing a company culture of mutual and cross-hierarchal support may be a key factor to promote EMS personnel’s health in the long-term.

### Strengths and Limitations

Incorporating experienced EMS professionals to develop the checklist allowed integrating occupation-specific knowledge to revise and extend the existing checklist. By that, we were able to integrate the occupation-specific knowledge and perspective of key informers of the target population. Another strength of this study is, we investigated both mental and physical trauma-related stress symptoms, of which the latter were often disregarded in previous research (Sterud et al., 2006; cf. Halpern et al., 2009). The prevalence of mental disorders were comparable to previous studies (Donnelly, 2012). Additionally, the response rate (46.6%) in our study was considerably higher than in previous research. Admittedly as we have used convenience sampling, our sample might have differed from the population in unknown ways (“non-response bias”). Nevertheless, in regard to relevant socio-anagraphic characteristics, the recruited sample corresponded well with the entire work population of the local German Red Cross ambulance service stations. EMS personnel who were confronted with particularly high doses of stress and trauma could have been currently or chronically ill, resigned from their jobs or prematurely retired from it. Due to their absence event prevalence and its effects might have been underestimated (“healthy worker effect”; Costa, 2003). Constraints on generality may result from the fact that checklist development and empirical findings are based on the experiences of EMS personnel responsible for Central European provincial towns with comparatively high income, good infrastructure, and low crime rate. The type and frequency of emotionally stressful mission events could considerably differ among populations depending on their respective socio-cultural, religious or geographic backgrounds. The checklist can be considered as a living document that may be expanded with regionally specific items such as natural disasters (e.g., earthquakes, floods), singular disasters (e.g., nuclear accidents) or compromised public security (e.g., missions in civil war zones, in areas with rampant violent crime and/or restricted or lacking statehood). Further important, our results are to be interpreted correlational due to the cross-sectional and retrospective study design. Longitudinal studies are necessary to further confirm the predictive validity of the proposed checklist scores for the incidence of mental and physical stress symptoms. Finally, participants’ response behaviour might have been biased by social desirability. There is evidence that expressions of distress and stress-related health complaints are often concealed in the EMS (Häller et al., 2009; Halpern et al., 2009). However, an employed social desirability questionnaire (SDS-CM short scale; Lück and Timaeus, 1997) indicated no such response behaviour.

## Conclusion

Rescue missions can be traumatic (i) if EMS personnel encounter threats or incur serious injuries; (ii) if certain circumstances cause personnel to give up their professionally detached perspective on patients; (iii) if they perceive missions as exceptionally tragic. The corresponding items of the newly introduced RESQ checklist showed the highest correlations with post-traumatic, depressive and physical stress symptoms among our study cohort. Therefore, the sum score of these items (RESQ-CE) could present a suitable measure for quantifying the cumulative exposure to critical events in EMS rescue missions. Replications in independent samples are necessary to verify the validity of the sum score. Future studies could also investigate the combined effects of critical events encountered at work as well as in private life, and its impact on EMS personnel’s quality of life. This also demands investigating the possible detrimental effects of repeated duty-related trauma exposure on the supportive social network of EMS personnel.

## Data availability statement

The datasets for this manuscript are not publicly available because the data may not be passed on or published to third parties outside the research project. The dataset contains sensitive personal and clinical information that might allow identifying individual participants. We do not have the ethics committee’s or our participants’ consent to grant access to the collected data.

## Ethics Statement

The studies involving human participants were reviewed and approved by the Ulm University Ethics Committee. The patients/participants provided their written informed consent to participate in this study.

## Author Contributions

AB, RR, and I-TK developed the study concept and study design. RR coordinated the focus group for checklist revision and extension. AB collected the data with support of SK. AB performed data analyses. AB drafted the manuscript with support of SK and MH with RR and I-TK providing critical revisions. All authors read and approved the final manuscript.

## Conflict of Interest

The authors declare that the research was conducted in the absence of any commercial or financial relationships that could be construed as a potential conflict of interest.
