# Supplementary material for: Deconstructing Traumatic Mission Experiences: Identifying Critical Incidents and Their Relevance for the Mental and Physical Health Among Emergency Medical Service Personnel
Source: Front Psychol. 2019 Oct 22;10:2305. doi: 10.3389/fpsyg.2019.02305 (PMC6817588; doi:10.3389/fpsyg.2019.02305)
Supplement: Supplementary file 1 [file Data_Sheet_1.PDF]

Supplementary Material

Deconstructing traumatic mission experiences: Identifying critical incidents and their relevance for the mental and physical health among emergency medical service personnel

Alexander Behnke, Roberto Rojas*, Sarah Karrasch, Melissa Hitzler, Iris-Tatjana Kolassa

*** Correspondence:** Dr. Roberto Rojas, roberto.rojas@uni-ulm.de

Supplementary Table 1. Comparison of participants with complete and incomplete responses

	**Participants with complete data (*n* = 102)**	**Participants with incomplete data (*n* = 13)**	**Test statistic**
Age (years)	*Mdn* (*IQR*) = 26 (17)	*Mdn* (*IQR*) = 25 (8)	$U = 560.5, z = -0.91, p = .364, r = -.08$
Sex	male: *n* = 66 (64.7%)<br>female: *n* = 36 (35.3%)	male: *n* = 6 (46.2%)<br>female: *n* = 7 (53.8%)	$\chi^2(1) = 1.69, p = .193, V = .12$
Work experience (years)	*Mdn* (*IQR*) = 3.3 (10.9)	*Mdn* (*IQR*) = 4.3 (6.0)	$U = 571.0, z = -0.81, p = .416, r = -.08$
Work location	Ulm: *n* = 72 (70.6%)<br>Heidenheim: *n* = 30 (29.4%)	Ulm: *n* = 8 (61.5%)<br>Heidenheim: *n* = 5 (38.5%)	$\chi^2(1) = 0.45, p = .504, V = .06$
Qualification	Notfallsanitäter[a]: *n* = 61 (59.8%)<br>Rettungssanitäter[b]: *n* = 30 (29.4%)<br>Apprenticeship Notfallsanitäter: *n* = 11	Notfallsanitäter[a]: *n* = 6 (46.2%)<br>Rettungssanitäter[b]: *n* = 5 (38.5%)<br>Apprenticeship Notfallsanitäter: *n* = 2	$\chi^2(2) = 0.64, p = .639, V = .09$

	(10.8%)	(15.4%)	
Employment form	salaried: *n* = 87 (85.3%)<br>voluntary: *n* = 15 (14.7%)	salaried: *n* = 10 (76.9%)<br>voluntary: *n* = 3 (23.1%)	$\chi^2(1) = 0.61$, *p* = .434, *V* = .07
Nightshifts (per month)[†]	*Mdn* (*IQR*) = 5 (3)	*Mdn* (*IQR*) = 5 (3)	*U* = 539.0, *z* = -1.06, *p* = .289, *r* = -.10
Rescue missions (per month)[†]	*Mdn* (*IQR*) = 10 (12)	*Mdn* (*IQR*) = 10 (10)	*U* = 596.5, *z* = -0.54, *p* = .592, *r* = -.05
Routine transport mission (per month)[†]	*Mdn* (*IQR*) = 10 (11)	*Mdn* (*IQR*) = 10 (6)	*U* = 597.0, *z* = -0.53, *p* = .594, *r* = -.05
LEC-5	*Mdn* (*IQR*) = 4 (141)	*Mdn* (*IQR*) = 3 (4)	*U* = 567.0, *z* = -0.85, *p* = .393, *r* = -.08
RESQ	*Mdn* (*IQR*) = 22 (6)	*Mdn* (*IQR*) = 22 (10)	*U* = 603.0, *z* = -0.53, *p* = .595, *r* = -.05
RESQ−CE	*Mdn* (*IQR*) = 6 (5)	*Mdn* (*IQR*) = 6 (6)	*U* = 655.0, *z* = -0.07, *p* = .943, *r* = -.01

Note: Frequency distributions of staff and sample were compared using Pearson χ^2-tests (applying a Yates correction for 2×2-tables) with Cramer's *V* as effect-size measure. Continuous variables were compared using Mann-Whitney *U*-tests with Cohen's *r* as effect-size measure ([†] one missing value). Comparison tests were not calculated for perceived social support, posttraumatic, depressive, and physical symptoms because less than six cases were available from the group of participants with incomplete data. The levels of qualification are comparable with [a] Emergency Medical Technician-Paramedic and [b] Emergency Medical Technician-Basic.
